# Maternal health care services utilization amidstCOVID-19 pandemic in West Shoa zone, central Ethiopia

**DOI:** 10.1371/journal.pone.0249214

**Published:** 2021-03-26

**Authors:** Kababa Temesgen, Negash Wakgari, Bikila Tefera Debelo, Belay Tafa, Getu Alemu, Fikadu Wondimu, Tolera Gudisa, Tolosa Gishile, Gurmesa Daba, Gizachew Abdissa Bulto, Bikila Soboka

**Affiliations:** 1 Department of Midwifery, College of Medicine and Health Sciences, Ambo University, Ambo, Ethiopia; 2 Department of Medical Laboratory Sciences, College of Medicine and Health Sciences, Ambo University, Ambo, Ethiopia; 3 Department of Public Health, College of Medicine and Health Sciences, Ambo University, Ambo, Ethiopia; 4 Department of Obstetrics and Gynecology, Ambo University Referral Hospital, Ambo, Ethiopia; 5 Ethiopian Public Health Institute, Health System and Reproductive Health Research, Addis Ababa, Ethiopia; University of Mississippi Medical Center, UNITED STATES

## Abstract

The novel coronavirus (COVID-19) is an infectious disease caused by a newly discovered coronavirus. Despite strong efforts that have been taking place to control the pandemic globally, the virus is on the rise in many countries. Hence, this study assessed the maternal health care services utilization amidst the COVID-19 pandemic in West Shoa zone, Central Ethiopia. A community-based cross-sectional study was conducted among 844 pregnant women or those who gave birth in the last 6 months before the study. A multi-stage sampling technique was used to select the study participants. The data were collected through face-to-face interviews using a semi-structured questionnaire. Logistic regressions were performed to identify the presence of significant associations, and an adjusted odds ratio with 95%CI was employed for the strength and directions of association between the independent and outcome variables. A P-value of <0.05 was used to declare statistical significance. The prevalence of maternal health service utilization during the COVID-19 pandemic was 64.8%. The odds of maternal health service utilization was higher among mothers who had primary (AOR = 2.16, 95%CI: 1.29–3.60), secondary (AOR = 1.97, 95%CI: 1.13–3.44), and college and above education (AOR = 2.89, 95%CI: 1.34–6.22) than those who could not read and write. Besides, mothers who did travel 30–60 minutes (AOR = 0.37, 95%CI: 0.23–0.59) and 60-90minutes (AOR = 0.10, 95%CI: 0.05–0.19) to reach the health facility had a lower odds of maternal health service utilization than those who did travel <30 minutes. Moreover, mothers who earn 1000–2000 (AOR = 3.10, 95%CI: 1.73–5.55) and > 2000 birrs (AOR = 2.66 95%CI: 1.52–4.64) had higher odds of maternal health service utilization than those who earn <500 birrs. Similarly, the odds of utilizing maternal health service were higher among mothers who did not fear COVID-19 infection (AOR = 2.79, 95%CI: 1.85–4.20), who had not had to request permission from husband to visit the health facility (AOR = 7.24, 95%CI: 2.65–19.75), who had practicedCOVID-19 prevention measure (AOR = 5.82, 95%CI: 3.87–8.75), and used face mask (AOR = 2.06, 95% CI: 1.28–3.31) than their counterpart. Empowering mothers and creating awareness on COVID-19 preventionis recommended to improve maternal health service utilization during the COVID-19 pandemic.

## Introduction

The novel coronavirus is a virus causing respiratory illness, commonly known as COVID-19 first noted in December 2019 in Wuhan, China, and has since then spread to countries in the world [1]. The World Health Organization (WHO) has declared the virus outbreak as a global pandemic on March 11, 2020 [2]. As of September 14, 2020, the virus has infected more than 28 million and killed more than 900,000 people [3]. Africa contributes 4% of infection (more than 1 million) and 3% deaths (more than 23,000) [3]. The WHO classifies maternal health services into antenatal care (ANC), delivery services, and postnatal care, as essential health services to continue during the COVID-19 pandemics [4, 5].

The maternal deaths were predicted to rise by 17% in the best-scenario and 43% in the case of the worst scenario due to the COVID-19 pandemics [6]. Movement restrictions, transport challenges, and anxiety over possibly being exposed to coronavirus are acting as the barrier to maternal health service utilization [6, 7]. The ongoing COVID-19 has affected the perceptions of pregnant women; more than half of the participants in a study done in Naples hospital reported the psychological impact of COVID-19 as severe, and a similar number of pregnant women to worry about the vertical transmission of the infection [7]. Strained health care systems, disruptions in care, and redirected resources might result in non-pandemic-related maternal morbidity and mortality and reproductive health crisis which are particularly true for African countries including Ethiopia with low-resource health systems [6, 8]. Obstetric healthcare providers may not be able to provide the highest quality care during the COVID-19 pandemic with essential precautions in place, client-provider communication is severely affected and the time to get the care needed may take longer as health workers try to protect themselves from the infection [9].

The International Confederation of Midwives and the Royal College of Midwives support community birth (home birth) for healthy women if there are appropriate midwife staffing and referrals are facilitated in obstetric emergencies, where these are not available, it may be necessary to modify available services, seeking at all times to maximize the provision of safe and positive birth experience to all women [4]. Ethiopia’s midwives grapple with COVID-19 while ensuring safe delivery, and maternal health workers reported that COVID-19 travel restrictions in Ethiopia are forcing pregnant women to give birth at home [8]. Maternity services in low resource countries are adapting to provide antenatal, delivery, and postnatal care amidst a rapidly shifting health system environment due to the COVID-19 pandemic [10]. Economic despair due to lost jobs, limited care, and restricted health services, overburdened health systems, restricted travel, and changing priorities at the primary care level are some of the burdens women had to face during the pandemic [11].

Many efforts have been taking place to enhance maternal health service utilization including information, education, and communication to raise awareness regarding the protection of mother and child during COVID-19, some countries have tried to open temporary birth centers, help hotlines virtual consultation with obstetricians have been provided via telemedicine services, to women seeking maternal health care service [6]. There is a consensus that the use of maternal health care services reduces maternal and child mortality and improves the reproductive health of women [12]. Family planning, ANC, use of skilled delivery attendants, and PNC services are maternal health services that can significantly reduce maternal morbidity and mortality. Since the novel coronavirus (SARS-CoV-2) is new to humans, only limited scientific evidence is available to identify the impact of the disease on maternal health service delivery and utilization particularly in low resource settings such as Ethiopia. Accordingly, understanding the factors that affect maternal health care services delivery and utilization amidst the COVID-19 pandemic could help to design appropriate strategies and policies towards the improvement of maternal health service provision and utilization. Thus, this study aimed to assess the maternal health care services (ANC, delivery, and PNC) utilization amidst the COVID-19 pandemic in West Shoa zone, central Ethiopia.

## Materials and methods

### Study design, setting, and population

A community-based cross-sectional study was conducted among women who were pregnant and those who gave birth in the last 6 months of the study period in West Shoa zone, central Ethiopia from July 1 to July 30, 2020. West Shoa zone is one of the zones of the Oromia National Regional state, its capital is located at114km distance to the western part of Addis Ababa, the capital city of Ethiopia. Currently, this zone has a total population of two and half million with around 1.2 million men and 1.3 women living within an area of 14,788.78 square kilometers. There are about 495,753 women of reproductive age and 100,283 mothers give births per year. West Shoa zone has 23 districts with over 528 rural kebele and 58 urban kebele. Currently, there are 8 Hospitals, 92 health centers, and 528 health posts in the West Shoa Zone.

### Sample size determination

The sample size was calculated using a single population proportion formula for the three maternal health services (ANC, Institutional delivery, and PNC). After calculating the sample size for the three maternal health services, the largest sample size was used as the overall sample size of the study. The assumptions considered during sample size calculation includes; 95% confidence interval, 5% the margin of error, and proportion of ANC visit 46% and proportion of institutional delivery 29% [13], and the proportion of early PNC visits 47% [14] in West Shoa zone from the previous study, and 10% non-response rate. The largest sample size calculated was for the proportion of early PNC visit 47%, n = 383, and used as the overall sample size of the study. The final sample size was 844 by considering a 10% non-response rate and the design effect of 2.

### Sampling techniques

A multi-stage stratified sampling technique was employed with strata of urban and rural kebele in the district. Six districts were selected at the first stage by simple random sampling from a total of 23 districts of West Shoa Zone. Then, kebele from the district was stratified into urban and rural kebele. Accordingly, one kebele from the urban and two kebeles from the rural were enrolled from the selected districts by simple random sampling. Mothers who were pregnant or gave birth in the last six months of the study were identified by doing a census in collaboration with health extension workers through the home-to-home visit. Then, a number of the study population was placed to each selected kebele using proportion to population size. After having the list of all women in each selected kebele, mothers were included using systematic random sampling techniques every interval of four.

### Data collection tools, procedure, and quality control

Data were collected using a semi-structured questionnaire through face-to-face interviews. The questionnaire was adapted from related literature and modified based on the objectives of the study and the cultural context of the study setting [3–8, 10, 11]. The tool consists of socio-demographic characteristics, maternal health care services amidstCOVID-19 pandemic (ANC, institutional delivery, and PNC) utilization, and impact of governmental emergency states (transport, distance, mask, and mandatory quarantine). Data collectors were 10 BSc health professionals, with health officers, Nurse, and Midwifery educational background. Pregnant mothers and those who gave birth in the last six months were identified first by using health extension workers to get their total number in the community. Data quality was assured during collection, coding, entry, and analysis. The two-day training was given for the data collectors and supervisors before actual data collection. The collected data were reviewed and checked for consistency, clarity; completeness, and accuracy throughout the data collection process by data collectors and supervisors. Furthermore, the pretest was done among 5% of the total sample in the Woliso district of the south-west Shoa zone to check clarity, general approachability, and feasibility of the questionnaire, and identify the specific problems of communication between the interviewer and the respondent. Moreover, the data collectors had utilized all necessary COVID-19 infection prevention measures such as face masks, sanitizer, and social distancing during data collection.

### Data processing and analysis

Collected data were cleaned and entered using Epi data version 4.6 and analyzed using SPSS version 23. Descriptive statistics were employed using frequencies, percentages, and diagrams. Multicollinearity among the independent variables was assessed by using variance inflation factors and no significant multicollinearity was detected. Both bivariable and multivariable logistic regressions were performed to identify the presence of a significant association between independent variables and the dependent variable. Those variables with a p-value of <0.2 in the bivariable analysis were considered for multivariable logistic regression analysis. Hosmer and Lemeshow goodness of fit test was used to check model fitness before running the final model. Finally, screened variables were fitted to the multivariable logistic regression model through a backward stepwise method to reduce the effects of cofounders and to identify the independent effects of each variable on the outcome variable. An adjusted odds ratio for a 95% confidence interval was employed for the strength and directions of association between independent variables and the outcome variables. A P-value of <0.05 was used to declare statistical significance.

### Operational definitions

Maternal health service utilization was defined as; received any ANC visit in line with their gestational age, or/and having institutional delivery, or/and any PNC visits during COVID-19. Those maternal health care services utilization were assessed using yes or no questions.

### Ethics statement

The ethical clearance was obtained from an ethical review committee of the college of medicine and health sciences, Ambo University. A letter of support was submitted to each district health office. Both written and verbal consent was obtained from each study subject before the data collection process proceeded. During the data collection process, the data collectors had informed each study participant about the objective and anticipated benefits of the research project and the study participants were also informed of their full right to refuse, withdraw, or completely reject part or all of their parts in the study.

## Result

### Socio-demographic and economic characteristics of the study participants

In this study, a total of 844 respondents were included; the response rate was 100%. Most of the subjects 669(79.3%) were in the 20–34 age group and 320(37.9%) of them had primary education. More than half, 467(55.3%) of the participants’ occupations were housewives. More than half of the study participant’s 467(55.3%) were housewives. Only one-third 303(35.9%) of the participants earn an estimated monthly income of greater than 2000 birrs (Table 1).

**Table 1:** Sociodemographic distribution of participants showing maternal health service utilization amidst the COVID-19 pandemic in West Shoa zone, central Ethiopia, 2020 (n = 844).

Characteristics	Frequency	Percentage
**Maternal age in years**		
< 19	43	5.1
20–24	229	27.1
25–29	242	28.7
30–34	198	23.5
35–39	102	12.1
≥40	30	3.6
**Marital status**		
Married	812	96.2
Others*	32	3.8
**Maternal education**		
Cannot read and write	165	19.5
Primary education	320	37.9
Secondary education	243	28.8
College and above	116	13.7
**Place of Residence**		
Urban	560	66.4
Rural	284	33.6
**Estimated monthly income in birrs**		
< 500	159	18.8
500–1000	200	23.7
1000–2000	182	21.6
> 2000	303	35.9
**Maternal occupation**		
Housewife	467	55.3
Government employee	117	13.9
Self-employed	146	17.3
Student	51	6.0
Farmer	63	7.5
**Husband occupation**		
Government employee	222	27.5
Self-employed	291	36.1
Student	19	2.4
Farmer	275	34.1
**Distance from the health facility**		
< 30 minutes	531	62.9
30–60 minutes	168	19.9
60–90 minutes	115	13.6
>90 minutes	30	3.6

*Single, divorced/widowed.

### Obstetric characteristics of the study participants

Among the 844 participants, 329(39.0%) were prim gravid and 657(77.8%) of them had 1–4 deliveries (parity) (Table 2).

**Table 2:** Obstetric characteristics of study participants to assess maternal health service utilization amidstthe COVID-19 pandemic in West Shoa zone, central Ethiopia, 2020 (n = 844).

Characteristics	Frequency	Percentage
**Previous adverse pregnancy outcome**		
Yes	165	19.5
No	679	80.5
**Gravidity**		
Prim gravida	329	39.0
2–4	468	55.5
≥5	47	5.6
**Parity**		
Null parity	165	19.5
1–4	657	77.8
≥5	22	2.6

#### Awareness of COVID-19 modes of transmission and prevention mechanisms

Among 844 of the participants, 835(98.9%) of them responded that COVID-19 can transmit from person to person and 230(27.3%) of them think they are at risk of getting the COVID-19 infection. Five hundred fifty-three, 65.5% were practicing at least two COVID-19 prevention measures (Table 3).

**Table 3:** COVID-19 related characteristics to assess maternal health service utilization among pregnant women and gave birth in the last 6 months of the study period in West Shoa zone, central Ethiopia, 2020 (n = 844).

Characteristics	Frequency	Percentage
**COVID-19 can transmit from person to person**		
Yes	835	98.9
No	9	1.1
**Handshaking with a person having the virus can transmit COVID-19**		
Yes	812	96.2
No	32	3.8
**Sitting nearby a person having the virus can transmit COVID-19**		
Yes	554	65.6
No	290	34.4
**Body fluid of a person having the virus can transmit COVID-19**		
Yes	294	34.8
No	550	65.2
**Cough of a person having the virus can transmit COVID-19**		
Yes	639	75.7
No	205	24.3
**Sexual intercourse with an infected person can transmit COVID-19**		
Yes	25	3.0
No	819	97.0
**At the risk of getting the COVID-19**		
Yes	230	27.3
No	614	72.7
**Confidence to protect from COVID-19**		
Yes	763	90.4
No	81	9.6
**Fear to visit the health facility**		
Yes	481	57
No	363	43
**Information about COVID-19 prevention**		
Yes	579	68.6
No	265	31.4
**Has sanitizer and/or alcohol**		
Yes	191	22.6
No	653	77.4
**Use of social distancing**		
Yes	526	62.3
No	318	37.7
**Use of face mask**		
Yes	609	77.2
No	235	27.8
**Practicing at least two of COVID-19 prevention measures**		
Yes	553	65.5
No	291	34.5
**Request permission from the husband to visit the health facility (n = 812)**		
Yes	735	90.5
No	77	9.5
**Media presence**		
Yes	722	85.5
No	122	14.5

### Reasons for not utilizing maternal health services

**Fig 1:**
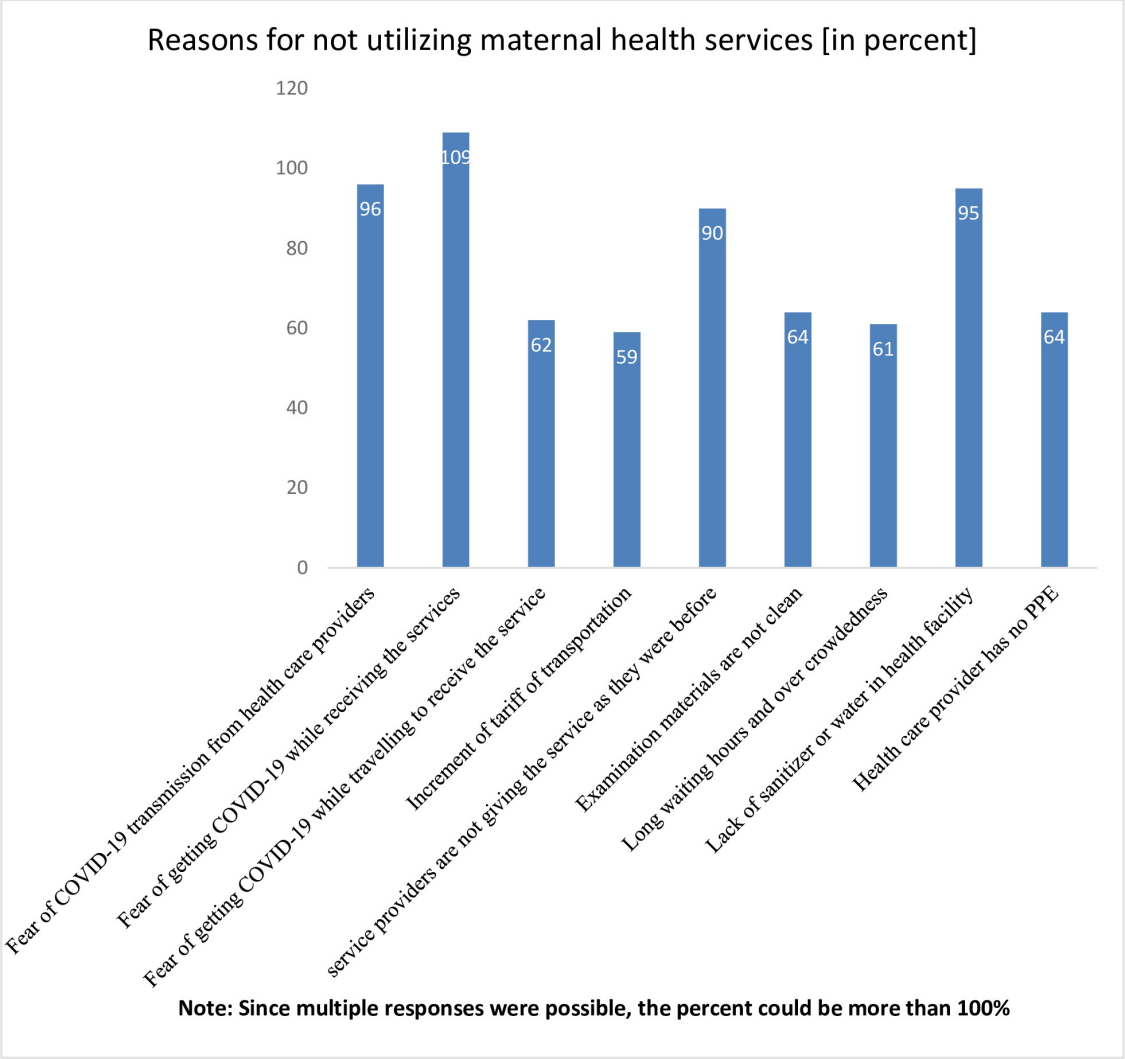
Reasons for not using maternal health service utilization during COVID-19, West Shoa zone, central Ethiopia, 2020.

Among the 297 participants who did not utilize maternal health services, 109(36.5%) mention fear of getting COVID-19 while traveling to receive the service as a reason to not receive the service, and 96(32.3%) and 95 (32%) gave fear of COVID-19 transmission from health care providers and lack of sanitizer or water in a health facility as a reason not to utilize the maternal health services respectively (Fig 1).

### Prevalence of maternal health service utilization and associated factors

In this study, about two-thirds 547(64.8%) [95% CI: 61.5–68.0] of mothers received maternal health care service during COVID-19. Maternal age, maternal occupation, husband occupation, educational status, place of residence, distance from the health facility, estimated monthly income, media presence, marital status, gravidity, parity, face mask use, fear of COVID-19 infections, permission request from husband to visit a health facility, sitting nearby a person having the virus can transmit COVID-19, cough of a person having the virus can transmit COVID-19, sexual intercourse with an infected person can transmit COVID-19, risk of COVID-19 infection, confidence to protect COVID-19, received information about COVID-19 prevention, and the practice of COVID-19 prevention measure had a p-value of < 0.2 in bivariable logistic regression and taken to the multivariable logistic regression. However, maternal educational status, distance from the health facility, monthly estimated income, fear of COVID-19 infection, permission request from husband to visit a health facility, and practicing COVID-19 prevention measures were significantly associated with maternal health service utilization in multivariable logistic regression.

The odds of maternal health service utilization during the COVID-19 pandemic in mothers who had primary education were 2.16 times higher than those who could not read and write (AOR = 2.16, 95% CI: 1.29–3.60). Similarly, mothers who had secondary education had higher odds of maternal health service utilization during the COVID-19 pandemic than those who could not read and write (AOR = 1.97, 95% CI: 1.13–3.44). Moreover, the odds of maternal health service utilization during the COVID-19 pandemic in mothers who had college and above education were 2.89 times higher than those who could not read and write (AOR = 2.89, 95% CI:1.34–6.22).

Mothers who did travel 30–60 minutes to reach the health facility were 63% less likely to utilize maternal health service during the COVID-19 pandemic than those who did travel <30 minutes to reach the health facility (AOR = 0.37, 95% CI:0.23–0.59). Furthermore, mothers who had to travel 60–90 minutes to reach the health facilities were 90% less likely to utilize maternal health services during the COVID-19 pandemic than those who did travel <30 minutes to reach the health facility (AOR = 0.10, 95% CI:0.05–0.19). While, mothers who earn 1000–2000 birrs had higher odds of utilizing maternal health service during the COVID-19 pandemic than those who earn <500 birrs (AOR = 3.10, 95% CI: 1.73–5.55). Likewise, the odds of maternal health service utilization during the COVID-19 pandemic were 2.66 higher in mothers who earn ≥2000 birrs than those who earn <500 birrs (AOR = 2.66, 95% CI: 1.52–4.64).

The odds of utilizing maternal health care service during the COVID-19 pandemic in mothers who did not fear COVID-19 infection was 2.79 times higher than in those who did fear COVID-19 infection (AOR = 2.79, 95% CI:1.85–4.20). Moreover, mothers who had not had to request permission from their husband to visit the health facility had 7.24 times higher odds of utilizing maternal health care service during the COVID-19 pandemic than those who had to request permission from their husband to visit the health facility (AOR = 7.24, 95% CI: 2.65–19.75). The odds of utilizing maternal health care services during the COVID-19 pandemic in those who practice COVID-19 prevention measures were 5.82 times higher than in those who did not practice the prevention measures (AOR = 5.82, 95% CI: 3.87–8.75). The odds of maternal health services utilization amidst COVID-19 were 2.06 times higher among mothers who use a face mask than those mothers who did not use a face mask (AOR = 2.06, 95% CI:1.28–3.31) (Table 4).

**Table 4:** Bivariable and multivariable logistic regression to assess factors associated with maternal health services in the amidst of the COVID-19, West Shoa zone, central Ethiopia, 2020 (n = 844).

Variables	Maternal health service utilization		Crude OR [95% CI]	Adjusted OR [95%CI]
	No	Yes		
**Maternal in years**				
< 19	15	28	4.36 (1.60–1.86)	3.03 (0.66–13.93)
20–24	75	154	4.79(1.09–10.97)	2.74(0.84–8.96)
25–29	68	174	5.97(1.60–13.69)	3.03 (0.71–9.05)
30–34	82	116	3.30 (1.02–7.57)	3.03 (0.62–9.05)
35–39	36	62	4.28(1.05–10.32)	4.16 (0.86–13.34)
≥40	21	9	1	1
**Maternal occupation**				
Housewife	192	275	1	1
Government employee	22	95	3.02(1.83–4.97)	0.61 (0.28–1.31)
Self-employed	42	104	1.73(1.16–2.59)	0.74 (0.42–1.30)
Student	22	29	0.92(0.51–1.65)	0.62 (0.29–1.36)
Farmer	19	44	1.62 (0.92–2.86)	1.97 (0.96–4.03)
**Husband occupation**				
Government employee	52	170	3.70(2.50–5.47)	0.98 (0.52–1.86)
Self-employed	67	224	3.78(2.64–5.43)	1.49 (0.84–2.64)
Student	9	10	1.26(0.50–3.19)	1.81 (0.45–7.24)
Farmer	146	129	1	1
**Educational status**				
Cannot read and write	105	60	1	1
Primary education	103	217	3.69(2.49–5.47)	2.16 (1.29–3.60)
Secondary education	71	172	4.24(2.78–6.46)	1.97 (1.13–3.44)
College and above	18	98	9.53(5.26–17.26)	2.89 (1.34–6.22)
**Place of residence**				
Urban	143	417	3.45 (2.56–4.67)	1.32 (0.73–2.40)
Rural	154	130	1	1
**Distance from the health facility**				
<30 minutes	117	414	1	1
30–60 minutes	87	81	0.26 (0.18-.38)	**0.37 (0.23–0.59)**
60–90 minutes	80	35	0.12 (0.08–0.19)	**0.10 (0.05–0.19)**
>90 minutes	13	17	0.37 (0.17–0.78)	0.53 (0.21–1.34)
**Estimated monthly income in birrs**				
< 500	87	72	1	1
500–1000	108	92	1.03(0.68–1.56)	1.15 (0.66–2.00)
1000–2000	45	137	3.68 (2.32–5.82)	**3.10 (1.73–5.55)**
> 2000	57	246	5.22 (3.41–7.98)	**2.66 (1.52–4.64)**
**Media**				
Yes	225	497	3.18 (2.15–4.72)	1.01 (0.55–1.86)
No	72	50	1	1
**Marital status**				
Married	269	528	2.89 (1.59–5.28)	1.96 (0.15–26.08)
Others*	28	19	1	1
**Gravidity**				
Prim gravida	95	234	3.63 (1.93–6.81)	1.51 (0.64–3.52)
2–4	174	294	2.49 (1.35–4.59)	0.96 (0.43–2.14)
≥5 pregnancies	28	19	1	1
**Parity**				
Null para	57	108	4.06 (1.57–10.53)	2.73 (0.53–14.00)
1–4	225	432	4.11 (1.65–10.24)	2.16 (0.49–9.57)
≥ 5	15	7	1	1
**Use face mask **				
Yes	109	126	1.94(1.42–2.64)	**2.06 (1.28–3.31)**
No	188	421	1	1
**Fear of COVID-19 infection**				
Yes	212	269	1	1
No	85	278	2.58 (1.91–3.49)	**2.79 (1.85–4.20)**
**Permission request from husband to visit the health facility**				
Yes	264	471	1	1
No	11	66	3.36 (1.75–6.48)	**7.24 (2.65–19.75)**
**Sitting nearby a person having the virus can transmit COVID-19**				
Yes	171	383	1.72 (1.28–2.31)**	0.87 (0.56–1.34)
No	126	164	1	1
** Cough of a person having the virus can transmit COVID-19**				
Yes	202	437	1.87 (1.36–2.58)	1.24 (0.79–1.93)
No	95	110	1	1
**Sexual intercourse with an infected person can transmit COVID-19**				
Yes	4	21	2.92(0.99–8.60)	1.90 (0.55–6.57)
No	293	526	1	1
**Risk of COVID-19 infection**				
Yes	53	177	2.20 (1.56–3.12)	1.52 (0.98–2.38)
No	244	370	1	1
**Confidence to protect COVID-19**				
Yes	262	501	1.46 (0.92–2.32)	1.69 (0.86–3.30)
No	35	46	1	1
**Received information about COVID-19 prevention**				
Yes	166	413	2.43 (1.80–3.29)	**0.71 (0.22–2.24)**
No	131	134	1	1
**Practice COVID-19 prevention measure**				
Yes	148	405	2.87 (2.13–3.87)	**5.82 (3.87–8.75)**
No	149	142	1	1

*Single, divorced/ widowed.

## Discussion

This study found the prevalence of maternal health service utilization was 64.8% [95%CI: 61.5–68.0]. This prevalence is lower than the studies done in Ethiopia before the COVID-19 pandemic [14, 15]. This might be due to the lockdown and movement restrictions brought about by the ongoing pandemic and lack of transportation manifested as a result [16]. Also, the dread and anxiety of visiting hospitals during COVID-19 has led many women to change their plan of childbirth and they are planning to have home deliveries [6].

The current study reported that the odds of maternal health service utilization during the COVID-19 pandemic were higher among mothers who had primary, secondary, and college and above education than those who could not read and write. This is consistent with the studies done in developing countries [17], and Nigeria [18]. Moreover, mothers who earn more than 1000 birrs per month had higher odds of utilizing maternal health services during the COVID-19 pandemic than those who earn < 500 birrs. This is in agreement with the studies done in developing countries [17], and Nepal [19]. This is because economically independent women do not rely on other people to decide to utilize maternal health services [20].

Furthermore, mothers who did travel 30–60 minutes and 60–90 minutes to reach the health facility were 63% and 90% less likely to utilize maternal health service during the COVID-19 pandemic than those who did travel <30 minutes to reach the health facility respectively. This might be due to a change of plan of ANC follow-up, childbirth, and other maternal health services hence the first delay, and planning to have home deliveries [6, 20]. Besides, the odds of utilizing maternal health service during the COVID-19 pandemic in mothers who did not fear COVID-19 infection was 2.79 times higher than those who did fear COVID-19 infection. This could be due to the psychological and social effects of the COVID-19 pandemic which is more pronounced in women forcing them to plan home deliveries not to expose their family to the infection [6]. Besides, mothers who had not had to request permission from their husband to visit a health facility had higher odds of utilizing maternal health service during the COVID-19 pandemic than those who had to request permission from their husband to visit a health facility. This is consistent with the studies done in Ethiopia [14, 21]. This might be due to women’s autonomy is positively associated with maternal health service utilization. Also, women with control over resources including physical, human, intellectual, and financial resources are more independent in deciding and seeking health care [21]. The odds of utilizing maternal health service during the COVID-19 pandemic in those who practice COVID-19 prevention measures was 5.82 times higher than in those who did not practice the prevention measure. Similarly, the odds of maternal health service utilization amidst COVID-19 were 2.06 times higher in mothers who use a face mask than those mothers who did not use a face mask. This is following the study done in Iran [22].

## Conclusion

In this study, the prevalence of maternal health service utilization was found low. Furthermore, maternal educational status, distance from the health facility, monthly estimated income, fear of COVID-19 infection, permission request from husband to visit a health facility, and practicing COVID-19 prevention measures were found to be significantly associated with maternal health service utilization. The federal ministry of health and regional health bureau should work in collaboration to aware of the community and mothers, in particular, to increase the maternal health service during the pandemic while practicing the necessary preventive measures to control the spread of the infection.

## Supporting information

S1 Questionnaire Afaan Oromoo version. (DOCX)

S2 Questionnaire English version. (DOCX)

## Abbreviations

ANC: Antenatal Care
COVID‐19: Corona Virus Disease 2019
PNC: Postnatal Care
WHO: World Health Organization
