# Supplementary material for: Maternal health care services utilization amidstCOVID-19 pandemic in West Shoa zone, central Ethiopia
Source: PLoS One. 2021 Mar 26;16(3):e0249214. doi: 10.1371/journal.pone.0249214 (PMC7997037; doi:10.1371/journal.pone.0249214)
Supplement: S1 Questionnaire — (DOCX) [file pone.0249214.s001.docx]

**Gaaffilee Qorannoo Itiyoophiyaa keessaati Goodina showaa lixaatti, Waa’ee tajaajila haadholii ulfaaf kennamu Fi weerara koronaa (Covid-19) Wajjin Qalqabatee kan Gaafatame, 2020.**

| **Adda baafannoo** | | |
| --- | --- | --- |
| - 1. **Kooddii adda baafannoo___________________** | | |
| - 1. **GG-JJ-WW __________________** | | |
| - - 1. **Gaaffilee haala hawaas-dinagdee hirmaattotaa ilaalchisee** | | |
| 1. Umirii | _________years | |
| 1. Haala gaa’eilaa | 1. heerumeera 2. hin heerumne 3. hiikeera 4. na irraa du’eera. | |
| 1. Bakkajireenyaa | 1. Magaala 2. Baadiyaa | |
| 1. Amantii | 1. Ortodoksii  2. Protestantii(peenxee)  3. Muusiliima  4. Kaatolikii  5. kan biroo(asittibarreessi)__________________________ | |
| 1. Sabnikee | 1. Oromoo 2. Amaara 3. Kan biro (asitti barreessi)__________________________ | |
| 1. Sadarkaa barumsaa | 1. kan hinbaranne 2. sadarkaa 1ffaa (Kutaa 1-8) 3. Sadarkaa 2faa (Kutaa 9-12) 4. Universiitii ykn koolleejii fi isaa ol 5. Hinbeeku | |
| 1. Hojiin kee maali dha? | 1. Haadha manaa 2. Hojjetaa mootummaa 3. Hojjetaa mit-mootummaa 4. Daldalaa 5. Barataa 6. Qoteebulaa 7. Hojii guyyaa (*qensiraa*) 8. Kan biroo(asittibarreessi)_________________ | |
| 1. Hojiin abbaa manaa kee maali dha? | - 1. Hojjetaa mootummaa   2. Hojjetaa NGO(mit-mootummaa)   3. Hojii dhuunfaa   4. Barataa   5. Qotee bulaa   6. Daldalaa   7. Hojii guyyaa (qensiraa)   8. Kan biro (asitti barreessi)__________________________ | |
| 1. Tilmaamaan Qarshii ji’aan argattu | 1. Qarshii <500 2. Qarshii 500-1000 3. Qarshii 1000-2000 4. Qarshii >2000 | |
| 1. Fageenyi mana kee fi mana yaalaa gidduu hammam ta’a? | 1. Deemsaan <30 daq 2. Deemsaan 30-60 daq 3. Deemsaan 60-90 daq 4. Deemsaan >90 daq | |
| **II. Gaaffillee waa’ee haala wal hormaata fayyaa ilaalchisee** | | |
| 1. Si’a meeqa ulfooftee turte? | | Ulfa_______________ |
| 1. Sia meeqa deessee turtee? | | Dahumsa________________ |
| 1. Mana yaalaa deemuuf jettee abbaa waarraa kee eeyyama gafattaa? | | - 1. Eeyee   2. lakki |
| 1. Abbaan warraa kees mana yaalaa siif eeyyamee deemi siinjedhee beekaa?? | | - - - 1. Eeyee       2. Lakki |
| 1. rakkina ulfa wajjin wal-qalqabatee kanaan dura qabdaa? | | - - - 1. Eeyee       2. lakki |
| \| **III. Gaaffilee yaallitii haadholiif keennamuu fi Weerara COVID -19 wajjin walqabatee** \| \| --- \| | | |
| 1. Odeeffannoo waa’ee koronaa(COVID-19) dhageesseetaa? | | - 1. Eeyee   2. Lakki----🡪gara gaaffii 18tti |
| 1. ‘Eeyyee’ yoojette (G-16), maal irraa dhageessee? | | 1. Radiyoo 2. Televijinii 3. Maatii 4. Ollaa 5. wal-gahii gandaa irratti 6. Hojjettoota fayyaa eksitenshinii 7. Hojjettootafayyaairraa 8. kanbiroo(asittibarreessi)__________________________ |
| 1. midiyaa qabduu? | | - 1. Eeyee   2. Lakki |
| 1. Koroonaan nama irraa namatti daddarba jettee yaaddaa? | | - 1. Eeyee   2. Lakki |
| 1. Koronaan harka wal dubbisuu/tuquudhaan daddarba jette yaaddaa? | | - - - 1. Eeyee       2. Lakki |
| 1. Koronaan wal bira taa’uu dhaan daddarba jettee yaaddaa? | | 1. Eeyee  2. Lakki |
| 1. Koronaan dhangala’oo qaama keenyaa bahuun daddarbuu daanda’aa? | | 1. Eeyee  2. Lakki |
| 1. Koronaan qufaadhaan daddarbuu danda’aa? | | 1. Eeyee  2. Lakki |
| 1. Koronaan wal qunnamtii saalaatiin daddarbaa? | | 1. Eeyee 2. Lakki |
| 1. Balaa koronaadhaan nan qabama jettee yaaddee beektaa? | | - 1. Eeyee   2. Lakki |
| 1. Koronaa of irraa ittisuuf ofiitti amanamummaa qabdaa? | | 1. Eeyee 2. Lakki |
| 1. Koronaa sodaatteettaa? | | 1. Eeyee 2. Lakki |
| 1. Koronaa of irraa ittisuuf,Sanitizerii ykn Alkoolii fayyadamtettaa? | | 1. Eeyee 2. Lakki |
| 1. Koronaa of irraa ittisuuf fageenya kee ni eegatta? | | - 1. Eeyee   2. Lakki |
| 1. Koronaa of irraa ittisuuf,Haguuggii afaanii fi funyaaniii fayyaadmtee beektaa? | | - - - 1. Eeyee       2. Lakki |
| 1. Ji’a afur darbe keessatti (erga koronaan Itiyoophiyaa keessatti argamee asitti), tajaajila yaalaa haadholiif kennamu argachuuf mana yaalaa deemteettaa? | | - - - 1. Eeyee       2. Lakki |
| 1. Yoo deebiin kee eyyee ta’e [Gaaffi31], tajaajila fayyaa haadholiif kennamu keessa isa kam fayyadamtee? | | 1. Tajaajilada’umsaduraa (Antenatal care) 2. Da’umsaqofaa (Delivery service) 3. Tajaajila da’umsa boodaa (PNC) |
| 1. Yoo deebiin “lakki” jette (tajaajila fayyaa fin fayyadmne tahe),maaliif sababiin isaa? | | 1. Hojjetaa fayyaa irraa koronaan natti darba jedheen waan sodaadheef 2. Yeroo na yaalan koronaan natti darba jedheen waan sodaadheef 3. Yeroon karaa adeemu koronaan natti darba jedheen waan sodaadheef 4. Kaffaltiin geeejibaa waan dabaleef 5. Hojjeettootni fayyaa akka duraa isaanii tajaajila namaaf hin godhan 6. Teessoon fi sireen irratti nama yaalan qulqulluu miti. 7. Namoota dabaree baay’ee fi heddummina waan qabuuf 8. Hir’inni sanitizerii ykn bishaan waan jiruuf 9. Hojjettootni fayyaa huccuu of irraa ittisan (personal protective equipment (PPE)) waan hin qabneef. |
| 1. Ji’aa furii asitti mana yaalaa deemteetta yoo ta’e,waa’ee koronaa of irraa ittisuu hojjettaa mana yaalaa irraa gorsa/barumsa fudhattaniittu? | | - 1. Eeyee   2. Lakki--------🡪gara gaaffii 37tti |
| 1. Eeyee yoo jette,gorsa/barumsa kana hojiiirra oolchiteettaa? | | - - - 1. Eeyee---🡪gara gaaffii 37tti       2. Lakki |
| 1. Lakki yoo jette, (barumsa isaan siif kennan hojiirra oolchuu maaliif dadhabde? *(deebiibaay’ee/tokkoool/ deebisuudanda’u)*. | | 1. Alkoolii ykn sanitizerii waan hin qabneeef  2. Bishaan dhiqannaaf yknsaamunaa waanhinqabneef  3. hojiinkoo akka koronaa ofirra ittisu na hin taasisu  4. mana tokko fi dhiphoo keessa maatii waajin waanan jiraadhuuf  5. of irraa eeguu waanan nuffeef  6. Kan biroo*(asittibarreessi)___________________________* |
| 1. Ji’aa furii asitti tajaaajila yaalii fayyaa haadholiif kennamu irratti ,haala ati barbaadduun si yaalanii ati itti gammaddeettaa? | | - - - 1. Eeyee       2. Lakki |
| 1. Lakki yoo jette maaliif? *(deebii baay’ee deebisuu danda’u)*. | | - 1. Hojjetaa fayyaa irraa koronaan natty darba jedheen waansodaadheef   2. Yeroo na yaalan koronaan natty darba jedheen waan sodaadheef   3. Yeroo karaaa deemu koronaan natty darba jedheen waan sodaadheef   4. Kaffaltiin geeejibaa waan dabaleef   5. Hojjeettootni fayyaa akka duraa isaanii tajaajila namaaf hingodhan   6. Sireen irratti nama yaalan qulqulluumiti.   7. Teessoon irrataa’an qulqulluumiti   8. Namoota dabaree baay’ee waanqabuuf   9. Hojjettootni fayyaa huccuu of irraa ittisan (personal protective equipment (PPE)) waan hin qabneef.   10. Haadholii baay’ee waliitihiiqaniiwaantaa’aniif (meetira 2 gadi)   11. Alkoolii ykn sanitizerii waan hin qabneeef   12. Kan biro *(asitti barreessi)___________________________* |

**Hirmaannaa keessaniif Galatoomaa!!!**
