# Supplementary material for: Maternal health care services utilization amidstCOVID-19 pandemic in West Shoa zone, central Ethiopia
Source: PLoS One. 2021 Mar 26;16(3):e0249214. doi: 10.1371/journal.pone.0249214 (PMC7997037; doi:10.1371/journal.pone.0249214)
Supplement: S2 Questionnaire — (DOCX) [file pone.0249214.s002.docx]

**Questionnaires on the study of Maternal health care services utilization in the amid of COVID-19 pandemic in West Shoa Zone, Central Ethiopia**

| **Identification** |
| --- |
| - 1. Individual identification code___________________ |
| - 1. DD-MM-YY __________________ |

| 1. **Maternal socioeconomic and demographic characteristics of respondents.** | | | |
| --- | --- | --- | --- |
| 1. Age in year | _________years | | |
| 1. Marital status | - 1. Married   2. Single   3. Divorced   4. Widowed | | |
| 1. Place of residence | 1. Urban 2. Rural | | |
| 1. Religion | - 1. Orthodox   2. Protestant   3. Muslim   4. Catholic   5. Other specify ___________ | | |
| 1. Ethnicity | 1. Oromo 2. Amhara 3. Other specify _____________ | | |
| 1. Highest educational level | 1. Illiterate 2. Elementary school(Grade 1-8) 3. Secondary/high school(Grade 9-12) 4. University/college and above 5. Don’t know | | |
| 1. Mother’s Occupation | 1. Housewife 2. Government employee 3. NGO employee 4. Merchant 5. Student 6. Farmer 7. Daily laborer 8. Other specify _________________ | | |
| 1. Husband’s occupation | - - - 1. Government employee       2. NGO employee       3. Private employee       4. Student       5. Farmer       6. Merchant       7. Daily laborer       8. Other specify______________ | | |
| 1. Family wealth index (Income) | 1. <500 ETB 2. 500-1000 ETB 3. 1000-2000 ETB 4. >2000ETB | | |
| 1. Distance (minute) of heath facility from home | 1. <30 min walking time 2. 30-60 min walking time 3. 60-90 min walking time 4. >90 min walking time | | |
| **II. Reproductive health Characteristics of respondents** | | | |
| 1. What is the total number of pregnancies you had in your lifetime? | | _____________________ pregnancies | |
| 1. How much delivery you gave birth? | | _____________________ deliveries | |
| 1. Do you request permission from your partner/husband to use maternal health services | | - 1. Yes   2. No | |
| 1. Did your partner support you to use maternal health service? | | - - - 1. Yes       2. No | |
| 1. Do you have previous adverse pregnancy outcome? | | - - - 1. Yes       2. No | |
| **III. Questions related to maternal health service utilization during COVID 19 pandemic** | | | |
| 1. Do you have any information about COVID-19? | | | - 1. Yes   2. No--🡪 Go to Q-18 |
| 1. If Yes (Q16), from where did you get the information? | | | 1. Radio 2. Television 3. Family 4. Neighborhood 5. Kebele meeting 6. Community health extension workers 7. Other health care providers 8. Others_________________ |
| 1. Do you have media exposure? | | | - - - 1. Yes       2. No |
| 1. Do you think COVID-19 can transmit from person to person? | | | - 1. Yes   2. No |
| 1. Do you think Hand shaking with a person having the virus can transmit COVID-19 | | | - 1. Yes   2. No |
| 1. Do you think Sitting nearby a person having the virus can transmit COVID-19 | | | 1. Yes 2. No |
| 1. Do you think body fluid of a person having the virus can transmit COVID-19 | | | 1. Yes 2. No |
| 1. Do you think Cough of a person having the virus can transmit COVID-19 | | | - - - 1. Yes       2. No |
| 1. Sexual intercourse with an infected person can transmit COVID-19 | | | 1. Yes 2. No |
| 1. Do you think that are you at the risk of getting the COVID-19 | | |  |
| 1. Do you have Confidence to protect from COVID-19? | | | - - - 1. Yes       2. No |
| 1. Did you Fear to visit the health facility? | | | 1. Yes 2. No |
| 1. Did you use sanitizer and/or alcohol as a COVID-19 prevention measure? | | | 1. Yes 2. No |
| 1. Did you practice social distancing as a COVID-19 prevention measure? | | | - 1. Yes   2. No |
| 1. Did you use face mask as a COVID-19 prevention measure? | | | 1. Yes 2. No |
| 1. In the last four months (after COVID-19 has reported in Ethiopia), have you used health facility for maternal health care services? | | | - - - 1. Yes       2. No |
| 1. If yes to Q 31; which maternal health service did you use in the last 4 months (during COVID-19)? | | | 1. Antenatal care 2. Delivery service 3. Postnatal care |
| 1. If No to Q 31”, why? (Multiple answers are possible). | | | - - - 1. Fear of COVID-19 transmission from health care providers       2. Fear of getting COVID-19 while receiving the services.       3. Fear of getting COVID-19 while travelling to receive the service.       4. Increment of tariff of transportation       5. The service providers are not giving the service as they were before.       6. Chairs and/Examination bed are not clean       7. Over crowdedness of mothers       8. Lack of sanitizer or water in health facility (HF)       9. Health care provider has no personal protective equipment (PPE) |
| 1. If you have visited health facility in the last four months, did you receive information about COVID-19 prevention? | | | 1. Yes 2. No--->Go to Q 37 |
| 1. If yes, are you practicing the education you received from the service provider? | | | - - - 1. Yes---> Go to Q 37       2. No |
| 1. If No, why do you think you can not apply the preventions measures? (Multiple answers are possible). | | | 1. I don’t have sanitizer/alcohol at home  2. I don’t have water facility to wash my hands regularly.  3. My profession does not allow me to apply the prevention measures  4. I live in a single room with many people  5. negligence  6. Other reason ____________ |
| 1. If you have received maternal health service in the last four months, did you satisfied with the service? | | | - - - 1. Yes       2. No |
| 1. If no, why? (Multiple answers are possible). | | | 1. Fear of COVID-19 transmission from health care providers 2. Fear of getting COVID-19 while receiving the services. 3. Fear of getting COVID-19 while travelling to receive the service. 4. Increment of tariff of transportation 5. The service providers are not giving the service as they were before. 6. Examination bed are not clean 7. Chairs are not clean 8. Long waiting hours 9. Health care provider has no personal protective equipment (PPE) 10. Over crowdedness of mothers 11. Lack of sanitizer or water in health facility (HF) 12. Others______________________ |

**Thank you very much for your participation!!**
